# ‘Change means sacrificing a good life’: perceptions about severity of type 2 diabetes and preventive lifestyles among people afflicted or at high risk of type 2 diabetes in Iganga Uganda

**DOI:** 10.1186/1471-2458-14-864

**Published:** 2014-08-21

**Authors:** Roy W Mayega, Samuel Etajak, Elizeus Rutebemberwa, Goran Tomson, Juliet Kiguli

**Affiliations:** Makerere University School of Public Health, P.O. Box 7072, Kampala, Uganda; Department of Public Health Sciences Karolinska Institutet, Stockholm, Sweden; Department of Learning, Informatics, Management and Ethics (LIME), Karolinska Institutet, Stockholm, Sweden

**Keywords:** Type 2 diabetes, Perception, Lifestyle, Obesity, Diet, Physical activity, Self-monitoring

## Abstract

**Background:**

Interventions for prevention of type 2 diabetes ought to be acceptable to target communities. We assessed perceptions about type 2 diabetes and lifestyle change among people afflicted or at high risk of this disease in a low income setting in Iganga Uganda.

**Methods:**

Twelve focus group discussions (FGDs) of eight participants each were conducted, balancing rural and peri-urban (near the Municipality) residence and gender. The FGDs involved people with suspected type 2 diabetes (based on fasting plasma glucose (FPG), people with suspected pre-diabetes and obese people with normal FPG. Content analysis was conducted.

**Results:**

Diabetes was perceived to be a very severe disease. Its severity was attributed to its incurability and its numerous health effects. Men were also concerned about reduced sexual performance. However, participants’ strong concerns about the severity of diabetes were not reflected in their perceptions about the risk factors and lifestyles associated with it. While people with diabetes perceive obesity as ‘sickness’, those without diabetes perceive it as a sign of ‘success’. Although participants are willing to change their diet, they mention numerous barriers including poverty, family size, and access to some foods. Because of their good taste, reduction of high risk foods like sugar and fried food is perceived as ‘sacrificing a good life’. Increments in physical activity were said to be feasible, but only in familiar forms like domestic work. An over-arching theme emerged that ‘lifestyle changes are viewed as sacrificing a good life’.

**Conclusions:**

Health promotion should target both community norms and individual awareness regarding obesity, physical activity and diet, and should address the notion that obesity and unhealthy foods represent a good life. Health educators should plan with clients on how to overcome barriers and misconceptions to lifestyle change, leveraging the pervasive perception of type 2 diabetes as a severe disease to motivate change.

## Background

Evidence is now un-equivocal that type 2 diabetes is on the rise in sub-Saharan Africa [1]. A major shift in the health systems of low income countries is therefore necessary, to integrate prevention for non-communicable diseases [2, 3]. Personal lifestyle factors to reduce risk of type 2 diabetes have been known for decades [4–6]. They include: Healthy diets, physical activity and regular check-ups on health parameters (body weight, blood sugar, blood pressure, blood lipids, and adherence to therapy) [7, 8]. Others are cessation of smoking and harmful alcohol intake [6]. Evidence shows that these measures are efficacious especially if targeted to high risk persons [9]. However, the bulk of this evidence is from high income countries. Results from some of the few pilots in sub-Saharan Africa (Cameroon and South Africa) indicate that appropriate NCD programmes can be implemented in sub-Saharan Africa by optimizing human resources and health service delivery in culturally relevant ways [10, 11].

The success of individual-targeted lifestyle interventions depends on realistic goal setting [12, 13], yet current approaches to health education are often provider centred [5, 14, 15]. In their book ‘Sick Societies’ , Stuckler et al. note that ‘societies in which people are born, live, work and age create the individual’s risks for chronic diseases, for which individuals have little choice’ [6]. This implies that the normative context and environmental factors play a key role in determining an individual’s behavioural exposures, with the individual having little control over these factors. Because individuals are influenced by societal norms [16], lifestyle measures ought to be relevant to the context in which they are applied [17]. While the recommended behaviours are well known, there is inadequate information on forms of these behaviours that are feasible within the normative contexts of communities in sub-Saharan Africa [18].

Although nation-wide data on the prevalence of type 2 diabetes are not yet available for Uganda, some locality specific studies show substantial prevalence of the problem in some communities. A crossectional study in Kampala and Mukono Districts in 2002 estimated the prevalence of type 2 diabetes to be 8% among people aged ≥35 years [19]. A recent study among people aged 35–60 years in our study district showed a prevalence of type 2 diabetes of 7.4% [20]. A related study in the same context shows substantial prevalence of some risk factors for type 2 diabetes especially overweight (18%) and hypertension (21%) [21]. However, these studies lacked descriptive information on how individuals and households perceive diabetes related risk factors and how they can integrate recommended behaviours into their day-to-day lives. Four exploratory studies in Africa (including one in Eastern Uganda) focused on awareness about type 2 diabetes and its behavioural risk factors [11, 22–24] but their scope did not include community perceptions about preventive behaviours. There is a lack of studies that examine contextual factors and perceptions about preventive behaviours for diabetes in Uganda. As an important part of the health system, health educators should be guided on the forms of recommended lifestyles that are relevant to the community norms in their specific cultural settings.

Our objective was to describe perceptions about the severity of type 2 diabetes and views about the recommended preventive lifestyles among community members afflicted or at high risk for type 2 diabetes in a rural and peri-urban setting in Africa. We focused on how people perceive the feasibility and practicability of the recommended preventive behaviours. Among the preventive factors, our study focuses on perceptions about obesity, diet, physical activity and self-monitoring of health, describing the forms of change that are feasible and barriers to their uptake. The information generated will facilitate context specific health education for prevention of type 2 diabetes.

## Methods

### Study setting, design and population

This study was conducted in the Iganga-Mayuge Health and Demographic Surveillance Site (HDSS) [25] in eastern Uganda, about 120 kilometres from Kampala the Capital. It has 13 peri-urban (near the Municipality) and 52 rural villages. The HDSS set up supports population level research including availability of a data-base of all households and trained research assistants who can trace respondents to their households. Data was collected in April and May 2013.

**Figure 1:**
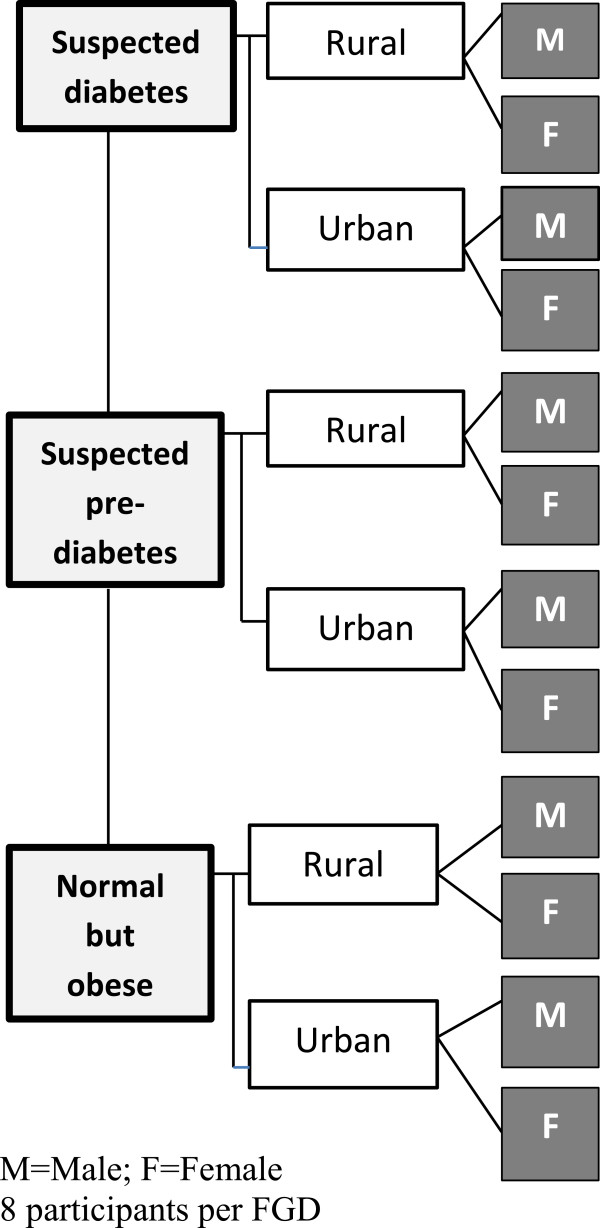
**Sample distribution for the FGDs.**

This was a qualitative study employing one of the ethnographic approaches, focus group discussions (FGDs). The FGD approach allowed participants to describe their feelings and perceptions on the issues for discussion [26]. The FGDs were homogenous with respect to age, sex and type 2 diabetes status, to allow free discussion with peers that share similar characteristics. A total of 12 FGDs were held, each comprising eight participants. This number of FGDs was selected to enable information saturation and comparison of perspectives from three main categories of participants: people with suspected type 2 diabetes (referred to in the rest of the article as ‘people with diabetes’), people with suspected pre-diabetes (referred to as ‘people with pre-diabetes’) and obese people with normal fasting plasma glucose (referred to as ‘obese’), stratified by gender and residence (See Figure 1). Suspected type 2 diabetes was defined on the basis of standard cut offs [27] as having a Fasting Plasma Glucose (FPG) level of ≥7.0 mmol/l, suspected pre-diabetes was defined as an FPG of 6.1-6.9 mmol/l, and normal Fasting Plasma Glucose as <6.1 mmol/l as determined in the prior survey referred to [28]. Obesity was defined using standard Body Mass Index (BMI) cut-offs as having a BMI > 30 Kg/m^2^ [29] as determined in the prior survey. Selecting participants from the prior study allowed easy identification of individuals from the three glycaemic categories mentioned. We use the term ‘suspected’ because the test used to classify diabetes status in the prior screening study (FPG) was non-confirmatory. All participants were aged between 35–60 years, an age-group that we have targeted in a series of studies due to its high prevalence levels of preventable risk factors. During analysis, evidence phenomenological saturation was supported by the observation that there were no new emergent themes by the 8^th^ FGD transcript.

### Sampling procedure

The sampling strategy was purposeful, targeting three categories of discussants as highlighted above. Participants were selected from those in a prior population survey that assessed prevalence of type 2 diabetes and pre-diabetes in the same area [21]. Using the database from the previous study, three lists were generated by sorting individuals in the specified categories with SPSS Version 18. Each list was then stratified into males and females (to create six categories) and into rural and peri-urban (to create 12 categories). Eight participants were then randomly selected from each sub-category using the SPSS 17 statistical software, to make 12 FGDs (See Figure 1). HDSS locator information was used to trace the sampled participants to their households to seek their consent. For six participants who declined to participate or were not found at their residence, replacements were sought using the same sampling approach. Consenting participants were invited to attend their respective FGD meeting.

### Data collection procedures

Two FGDs were held each day, one in the morning and the other in the afternoon. For each FGD, there was a moderator, an assistant moderator, an interpreter and a note-taker. Following greetings, verbal consent was obtained. Ground rules regarding participation were explained. The moderator and assistant moderator asked participants a series of open-ended and probing questions using a prepared FGD Guide. The note-taker audio-recorded the discussions while also taking notes and capturing the expressions of participants. The FGDs were moderated by the principal investigator (RWM) (a male public health specialist) assisted by a male co-moderator with experience in qualitative research (SE) to allow sufficient probing. They were supported by a female interpreter and a female note-taker, both conversant with the local language (Lusoga), the former being a nurse and the latter a social scientist. To reduce interference, the discussions were held in the Local Council halls at six selected sub-county offices.

To facilitate informed discussion on recommended lifestyles (diet, physical activity and self-monitoring), the moderators used an approach in which participants were told about the biomedical recommendations regarding a particular behavior and were then asked 'whether they were feasible and how’. This approach is similar to ‘motivational interviewing’ proposed by Miller and Rollnick [30] and recently evaluated by Witt et al. [31]. In this approach, ambivalent issues are clarified so that the focus is on ‘eliciting motivational processes that drive change’ [30]. For example, the moderators said: ‘*Experts advise that a healthy diet should balance energy giving foods like starchy food, with protein rich foods that build the body, vegetables or fruits and very little oil. Is this feasible for you?’.* The objective was to elucidate what it means to them to put the recommended behaviors into practice.

### The focus group discussion guide

The FGD guide used for this study covered perceptions on five issues: 1) Severity of type 2 diabetes, 2) obesity, 3) healthy diet, 4) physical activity, and 5) self-monitoring of health. The dimensions and questions used in the guide were derived from review of articles and guidelines on recommended lifestyles for prevention on type 2 diabetes from the WHO [32, 33]. The FGD guide was reviewed by the anthropologist (JK), and the health systems researcher (GT) on the team, who then discussed the interview items and probes with the field team. It was translated to the local language (Lusoga) and back-translated to English for accuracy.

### Data management and analysis

FGD recordings were labelled and stored. They were then transcribed and translated into English by an experienced research assistant fluent in both languages. All authors namely: RWM (a public health specialist), JK (an Anthropologist), SE (experienced in qualitative studies), ER (a public health specialist) and GT (a health system researcher) read the transcripts, and discussed emerging issues. Thereafter three investigators (RWM, JK and SE) developed a codebook. They did this by selecting three transcripts, re-reading them, assigning meaning units to each response and codes to each meaning unit while also taking notes on emerging sub-themes. They then met, combined their descriptive codes and discussed them to come up with a unified code book.

A content analysis approach was used, as described by Graneheim and Lundman [34] and applied by Nelson et al. [35]. All transcripts were entered into Atlas Ti, Version 5.2. Thereafter, each meaning unit in the transcripts was coded with the appropriate code from the codebook. Coding was conducted by two members of the research team (RWM and SE). Code concurrence between the two coders was found to me good (at 72%). The two coders met and harmonised the non-concurrent codes. After the coding, response units with similar codes were re-categorised into unifying sub-themes with the help of Atlas Ti. The sub-themes then guided the development of themes. The categories were then interpreted for their descriptive meaning. The authors also identified descriptive quotes from the content that represented key themes. An over-arching descriptive theme was then derived from the themes [34].

### Validity

A number of methodological approaches were used to promote validity. The inter-disciplinarity of the study team brought different perspectives into the study. Follow-up questions and probes were used to validate responses during the interviews. The moderators checked repeatedly with participants to ascertain assertions and meanings. Four coders developed the analysis code-book to enhance its reliability. The face-to-face rapport with the participants promoted trustworthiness.

### Ethics statement

This study was approved by Makerere University School of Public Health Ethics Committee (30^th^ June 2011), the Swedish Regional Ethics Board (Stockholm) (Diary Number 2010/2049-31/2) and the Uganda National Council of Science and Technology. Permission was also obtained from the Iganga-Mayuge HDSS management. Each participant consented to be involved, and anonymous identifiers were used in the discussions.

## Results

### Sample characteristics

A total of 96 participants were involved in the study, distributed in 12 focus groups. All participants were aged 35–60 years. Participants were in three categories: People with type 2 diabetes, people with pre-diabetes and obese people with normal blood sugar readings. Six of the FGDs were from rural areas while the other six were peri-urban. Six of the FGDs comprised of females and the others were male. A summary of the socio-demographic characteristics of participants is presented in the Table 1. Results are presented in five thematic topics as discussed in the FGDs. A summary of the key results is presented in Table 2.

**Table 1:** **Demographic characteristics of participants**

Characteristic	No (%)
Sex:	
Males	48 (50%)
Females	48 (50%)
Location of residence:	
Rural	48 (50%)
Peri-urban	48 (50%)
Age-group:	
35-39	32 (33%)
40-44	24 (25%)
45-49	18 (19%)
50-54	12 (13%)
55-60	10 (10%)
Main occupation:	
Subsistence farmer	60 (62%)
Trader	18 (19%)
Mechanic/Mason	11 (12%)
Formal/Salaried	07 (07%)
Highest level of education:	
None	19 (20%)
Lower Primary	20 (21%)
Higher Primary	35 (36%)
Secondary	17 (18%)
Tertiary	05 (05%)

**Table 2:** **Summary of results: emerging sub-themes and themes by participant category**

Questions	Category of participants	Results		
		Emerging sub-theme	Emerging theme	Over-arching theme
• Perceptions about severity of type 2 diabetes	**With suspected type 2 diabetes**	• Too much weight is sickness	*To persons with suspected diabetes obesity and eating ‘high risk foods’ signifies sickness; to those without diabetes obesity and ‘high risk foods’ signify is a good life*	**Lifestyle changes while possible involve sacrificing a good life i.e. there is an ‘opportunity cost’ to lifestyle change**
		• The ideal body weight is ‘medium’ – not too big and not too small		
• Perceptions about obesity				
• Perceptions about healthy diet				
• Perceptions about physical activity				
• Perceptions about monitoring their health	**With suspected pre-diabetes**	• Weight loss means sickness		
		• Obesity and ‘eating high risk foods’ signifies a success and a good quality of life		
		• Changing our diet means sacrificing a ‘good life’ and diet change should be gradual		
	**Obese but normo-glycaemic**			
	**All groups regardless of glycaemic status**	• Diabetes is very severe disease because it disables and is incurable	*Diabetes is a very severe disease*	
		• Diabetes is a very severe disease because of its complications/consequences		
		• Diabetes is a very severe disease because it weakens a man’s manhood		
		• Physical activity can be adjusted within what we already do but it should be gradual	*We are able to adjust physical activity, but in ways familiar to us*	
		• Some forms of physical activity are not feasible in our context		
		• It is possible to adjust our diet	*We can adjust to healthier diets but we are limited by important barriers*	
		• There are many barriers to a healthy diet		
		• It is possible to weigh ourselves regularly	*We can seek our health status periodically but we are sceptical about availability of services*	
		• There are many barriers to facility health check-ups		

### Perceptions on severity of type 2 diabetes

Participants from the majority of the FGDs viewed type 2 diabetes as a ‘very severe disease’. One category said it is severe because it ‘cannot be cured’ while others cited its consequences to affected people. Incurability of type 2 diabetes was qualified through assertions that ‘it kills’ , requires ‘life-long treatment’, disables, and that even the best treatments fail. They explained that people with diabetes require daily ‘injections’ and are forbidden from eating some foods.

*“Diabetes is a very severe disease. It weakens you, disables you, and then kills you. I have never seen a person with diabetes who has cured.”* (Male FGD, with diabetes, rural)

*“Diabetes will require you not to eat certain types of foods.... You always first have to inject yourself then you eat. If you miss treatment for a day, you can even die. Even if you treat it, even if you have lots of money, it can still kill you* (Female FGD, obese, rural)

Among participants who labeled type 2 diabetes as a very severe disease because of its consequences, there was a distinct gender difference in the types of consequences mentioned. Female participants were concerned about wide-ranging health effects of the disease. On the other hand, male participants were more concerned about the effect of type 2 diabetes on sexual performance, or ‘manhood’, which often resulted in marital stress.

*“Diabetes leads to loss of eye sight, heart disease, wounds all over the body, general weakness, pain all the time and no peace”* (Female FGD, with pre-diabetes, rural)

*“Diabetes weakens a man’s manhood, making him impotent or infertile. The wife might run away or cheat on the man”* (Male FGD, with diabetes, rural)

Both male and female FGD participants were equally concerned about the effect of diabetes on the family, especially loss of income. This concern was predominant in the rural FGDs.

*“When it affects the head, the whole family gets affected because this person becomes too weak to look after them. You also spend a lot on treatment”* (Male FGD, obese, urban)

### Views about body weight and obesity

There was a marked difference in perceptions about obesity between FGDs of people with suspected type 2 diabetes and those without. Participants with suspected type 2 diabetes viewed obesity negatively, associating it with disability, heart diseases and other chronic diseases.

*“Before I gained this weight I had no problem; now I have many complications – (high blood) pressure and joint problems. When someone is overweight, her energy and ability reduce. Weight is sickness”* (Female FGD, with diabetes, peri-urban)

This category of participants argued that associating obesity with wealth is a thinking of the past. Asked about the ideal body size, majority of people with diabetes described it as *‘medium - not too big and not too small’* and *‘weight that allows you to do simple chores like washing or lifting a can of water’*. However, the pejorative views about obesity were mainly expressed in the FGDs of people with suspected type 2 diabetes. Among participants without diabetes, obesity is associated with ‘being well-off’: ‘eating well’ , ‘having no worries’ and ‘high social standing’. They linked these factors to ‘success’, ‘peace of mind’ and ‘contentment’. The local word for an obese person (‘omuba’) also means ‘a very important person’.

*“In Busoga here when someone is big, they think that person has lots of money and no worries. They call them ‘omugaiga’ (the rich one) or ‘Byandaala’ (the well-off); if you are a woman, they call you ‘Hajjati’ (a show of respect). When you go to a function or the church they give you a front seat”.* (Female FGD with pre-diabetes, rural)

Some participants said that weight gain was inevitable with age, or if one has ‘large bones’. Some female participants attributed weight gain to use of contraceptives and menopause.

*“However physically active you are, when you are destined to grow fat, the weight simply comes. It runs in families; there is nothing you can do. Every morning I go to the garden; but I keep gaining weight…..”* (Male FGD, obese, peri-urban)

Participants from a majority of the FGDs said that weight loss is stigmatized. When one loses weight, people think he/she is very sick, suffers from HIV or is stressed due to loss of wealth.

*“If you are big and you lose weight people think you have HIV. When they see someone losing weight they say ‘that person is sick’.* (Female FGD, with pre-diabetes, rural);

*“If you lose weight people think you no longer have money. We now have micro-finance - people may think that you have failed to pay a loan…”* (Male FGD obese, peri-urban)

### Perceptions about healthy diet

Consistently across all FGDs, participants describe a healthy diet as eating ‘healthy foods’. However, to rural participants ‘unhealthy foods’ mean ‘sweet or sugary foods’, while peri-urban participants cite both sugary foods and ‘fried foods’ as unhealthy. Peri-urban participants widely believed that as long as meat is only boiled but not fried, its fat content is low. There was also a widely held view among rural participants that because of their sweet taste, sweet-potatoes are a high risk food, yet they are the staple food in the region. Although most peri-urban participants said that a balanced diet involves eating food from several food groups, rural participants considered it to be ‘eating any combination of foods’ regardless of the food groups. A few rural participants held the misconceptions that a balanced diet meant ‘eating soft foods’, which they described to mean *‘chewing food properly’* or *‘eating mashed food’*.

Participants were concerned about several barriers to a healthy balanced diet. Some said that cessation of high risk foods is not practical because they have the best taste. They said that the key sources of good taste in food were sugar, oil and salt. Large families, poverty and inability to afford some foods (especially protein rich foods) were cited as key barriers to a balanced diet.

*“The things they advise against are also the tastiest - it is difficult to eat un-fried beans. Without sugar or salt, you can’t enjoy life”* (Female FGD, with pre-diabetes, peri-urban)

*“Only when you have money can you eat chicken, fish and eggs; you cannot buy only half a kilo of meat to serve ten people”* (Male FGD, with diabetes, Urban)

Regarding access to food, majority of FGDs said that a range of foods are available in their region including various grains and root tubers as sources of starchy food, various pulses, small fish, meats and seed-oils for proteins and a wide range of vegetables and fruits, some growing in the wild. However, most FGDs said that it was not possible for each household to produce all the necessary foods for a balanced diet. Peri-urban dwellers buy most of their food.

*“At times the main food we grow is sweet potatoes. We face a very big problem finding the money to buy other food; we do not have banana or rice plantations. Sometimes all you have is maize flour.”* (Female FGD, with diabetes, rural)

Several participants said that adjustments to healthier diets are feasible. However, there was a consistent view that ‘change involved sacrificing a good life’ and replacing it with ‘a life of rules’). ‘Eating what one loves’ and ‘eating tasty food’ was viewed as ‘having a good life’. Most participants felt that although change was possible, it has to be gradual.

*“Change means sacrificing a good life…leaving foods that you enjoy. It is only possible if you reduce gradually.”* (Male FGD, with pre-diabetes, peri-urban)

### Perceptions about physical activity

Participants defined physical activity as ‘not sitting in one place’ but ‘keeping busy’. Across most FGDs, physical activity was viewed as ‘informal day-to-day activities’ rather than organized exercises. The majority said that the most feasible form of physical activity adjustment was by increasing domestic work. The range of possible chores suggested was diverse, including gardening, brick making, tending to domestic animals, fetching water, splitting firewood, bush clearing, and pounding food for rural areas, and washing, cooking, walking or cycling in peri-urban areas. There was a consistent view that physical activity increments should be nuanced on amplification of familiar forms of work, than taking up totally new activities.

*“Change is possible but in ways familiar to us. Everyone is involved in some domestic work;….the main issue is how much you do…”* (Male FGD, with diabetes, rural)

Most participants believe that programmed running/jogging is not feasible for adults in their context, unless it was part of other routine chores.

*“It’s impossible for people to wake up and run. Apart from sportsmen, I have never seen anyone leave his home to go running! May be the children; but for an adult to run…!”* (All laugh) (Male FGD, with pre-diabetes, peri-urban)

Several participants said that it is feasible to achieve 30 minutes of moderate physical activity daily. However, there was a consistent view that physical activity increments had to be gradual because they were not used to them. They said that initiating and sustaining physical activity increments requires self-drive and it meant a major adjustment in their lives.

### Perceptions about monitoring personal health

A majority of participants said that it was possible to regularly monitor their health. However, there were different interpretations of how this can be done. Some said they could monitor ‘how they feel’, and that ‘one could feel that he/she is unhealthy’. Others said they could weigh themselves regularly while others said they could go to the health facilities for checkups, some on a monthly basis and others every three or six months. Some barriers to going for health facility check-ups were cited though including transport, long distances, work commitments, and not knowing what a health check-up entails. Facility related concerns included: waiting times, service charges, fear of discovering ‘health problems’ that one could tolerate if they did not know about them and uncertainly about whether they would get the service when not ill.

*“When we are ill, health workers only ask questions and prescribe medications hurriedly without tests; how will they test us when we have no illness?”* (Female FGD, obese, rural)

## Discussion

Our findings show a disparity between participants’ perception of type 2 diabetes as a very severe disease compared to the urgency to take up preventive behaviours. We elucidate several perceptual and normative barriers to lifestyle change. While this phenomenon is not uncommon globally, our study adduces evidence that life conditions that are bio-medically regarded as high risk are on the contrary associated with wellness and ‘a good life’.

There is widespread perception of type 2 diabetes as a very severe disease. Whyte highlights that although NCDs are only emerging in Africa, awareness about their effects is ‘contagious’ as a result of interactions within family members and passive experiences with the health system [18]. Two consistent sub-themes underlying some explanations given for the severity of diabetes were the ‘futility of treatment’ and ‘assertion of chronicity’ referring to the endless injections and the incurability of the disease. Injections are often viewed as more potent and curative in many settings in rural Africa, and their failure to cure disease causes anxiety. Gender differences in concerns were observed. Women are concerned about the broader consequences of type 2 diabetes while men are more concerned about reduced sexual performance. Regardless of the reasons, the widespread perception that diabetes is a severe disease offers an entry point for ‘persuasion approaches’ to health education [36].

Although participants viewed diabetes as a very severe disease, the same was not reflected in their perceptions about key risk factors and lifestyles associated with preventing it. One of these is the stark difference in how people without diabetes perceive obesity. To persons with diabetes ‘obesity is sickness’ but to the majority without diabetes ‘obesity is success’. To convince an obese person who feels like she/he is ‘having the best of life’ to reduce weight is an enormous challenge, especially in clarifying what constitutes health. Two studies, one in the Middle East and another in the United Kingdom (UK) also showed a lower risk perception about obesity among overweight persons [37, 38] but contrasting findings were shown in a study among minorities in America [39, 40]. Studies in Africa have demonstrated a social desirability for overweight especially among women (‘big is beautiful’) [4, 41–43]. Weight loss is stigmatized and associated with diseases like HIV/AIDS, a finding highlighted in other studies in Africa [24, 44]. Several studies show that social norms strongly influence healthy lifestyles [45, 46]. Multilevel interventions that address ‘normative beliefs’ are therefore necessary [47].

Our study participants said that it is feasible to change to healthier diets but they are limited by several barriers including affordability of some foods and family size. What people perceive as un-healthy foods are consistent with those from two studies in Africa [22, 24] but rural-peri-urban differences were noted. Rural dwellers mention sugary foods while peri-urban dwellers mention both sugary foods and fried foods as high risk foods, a difference probably attributed to differences in level of awareness. There were also a number of misconceptions regarding diet including the belief that sweet potatoes are high risk foods (among rural folk) and that boiled meat has low fat content (among peri-urban participants). None of the FGDs mention ‘refined starchy foods’ like polished rice or maize flour as unhealthy, despite the increasing access to such foods in rural Africa [48]. Perhaps the most challenging misconception regarding a healthy diet is that communities link high risk foods to ‘enjoyment of life’. Overcoming these misconceptions should be prioritized in health education.

Our participants acknowledge that they are able to adjust their physical activity levels, but in familiar ways like domestic work. The range of chores suggested includes high intensity activities like gardening and brick making, moderate intensity activities (tending to animals, fetching water, splitting firewood, bush clearing) and low intensity (washing, cooking, pounding food and walking). These forms of physical activities are in contrast with preferences among urban residents in Cameroon, who viewed informal physical activities negatively [49], a discrepancy attributed to their urban context. Our findings also contrasted with those from two studies in North America where participants preferred structured exercises [50, 51], a difference attributed to norms regarding physical activity. Non-familiar activities like running are in our study context viewed as ‘strange’ , ‘laughable’ or ‘not suitable for adults’. Studies from the UK also reported embarrassment from formal gym activities or concern about ability to meet targets [16]. However, embarrassment in their setting was attributed to perceptions about their bodies other than from community censorship as observed in our setting.

Without self-monitoring, it is difficult to get an objective view of one’s health status. Indeed some studies have shown that the majority of obese persons deny, are not aware, or underestimate their condition [52–54]. Our study participants feel it is possible to monitor their health but some options they propose are un-objective, like basing one’s health status on ‘how someone feels’. While many barriers to seeking medical check-ups in health facilities were cited, three of the participants’ greatest concerns were the responsiveness of health workers to seemingly well people, not knowing what health ‘check-ups’ entail and fear of ‘unravelling what is not known’. These factors resonate with low confidence and other known barriers to access to health services in low income settings [55, 56]. With the many competing priorities in Uganda’s health system, establishment of wellness clinics or outreaches is a major challenge.

The mismatch between participants’ strong perceptions that type 2 diabetes is a very severe disease and their sense of urgency about behavior change raises important questions about the community’s ‘notion of health’. Many studies show that the main barriers to lifestyle change are insufficient knowledge about risk and lack of willpower [57–59]. Other studies found that individuals tend to externalize risk factors like diet and obesity as not attributed to their own lifestyle [45]. Our study adds a dimension to these studies by elucidating that life conditions regarded as high risk biomedically are instead viewed as indicative of ‘a good life’. The community’s notion of health is nuanced on their current wellness than future health. In at least two other studies, African participants have reported a positive outlook on life that discouraged thought about future sickness [42, 58]. There is need for ‘culturally sensitive’ health promotion in Africa that addresses barriers to change and a shift in paradigm to balance current and future health concerns [60] and in a way that promotes satisfaction with the process [61].

An over-arching theme emerging from our study is that ‘there is an opportunity cost to lifestyle change’ meaning that while lifestyle changes are viewed as possible, they are also regarded as ‘sacrificing a good life’– either from real or perceived barriers. On a positive note, people are willing to change provided solutions to the barriers are found and change is gradual.

## Conclusion

In conclusion, health educators should emphasize that diabetes is preventable with simple lifestyle changes that could easily be incorporated into everyday life and which do not mean ‘sacrificing the good life’. They should tactfully link the perceived severity of diabetes to its risk factors and preventive lifestyles, balancing perspectives on current and future health. Educators should also target the normative association of obesity and unhealthy foods to ‘a good life’. A social-ecological model that blends individual targeted health promotion with community approaches is necessary [56]. However, integration of preventive approaches requires some adjustments in the structure of health services to include population level prevention measures, wellness checks, and enhanced behaviour change communication for higher. This however is likely to present an enormous challenge to health systems that are already grappling with many challenges (human resources, finance, service delivery, diagnostics, therapeutics etc.).

### Methodological considerations

One of the limitations of this study is that participants were not on any formal lifestyle programs and therefore there was no discussion of motivators for change and intensity of lifestyle changes. The limitations of using a lead investigator from a biomedical background are acknowledged. However, the lead investigator had sufficient experience in FGDs, and was supported by an interdisciplinary team. Both the moderator and assistant-moderator were males, with the potential bias that women may not have opened up about their some of their concerns especially regarding sex. This was partly mitigated by using a female interpreter and female notes-taker, and sexuality was not a key research question for this study. The generalizability of our findings, while not statistical, applies to enriching our understanding by ‘zooming in’ into context specific perceptions about lifestyle change in settings similar to rural Iganga.

## References

[1] Dalal S, Beunza JJ, Volmink J, Adebamowo C, Bajunirwe F, Njelekela M, Mozaffarian D, Fawzi W, Willett W, Adami HO, Adami H, Holmes M (2011). Non-communicable diseases in sub-Saharan Africa: what we know now. Int J Epidemiol.

[2] Beaglehole R, Epping-Jordan J, Patel V, Chopra M, Ebrahim S, Kidd M, Haines A (2008). Improving the prevention and management of chronic disease in low-income and middle-income countries: a priority for primary health care. Lancet.

[3] Maher D, Smeeth L, Sekajugo J (2010). Health transition in Africa: practical policy proposals for primary care. Bull World Health Organ.

[4] Lagerros Y, Rossner S (2013). Obesity management: what brings success?. Therap Adv Gastroenterol.

[5] Schellenberg E, Dryden D, Vandermeer B, Ha C, Korownyk C (2013). Lifestyle interventions for patients with and at risk for type 2 diabetes: a systematic review and meta-analysis. Ann Intern Med.

[6] Stuckler D, Siegel K (2011). Responding to the global challenge of chronic disease, Oxford Univer.

[7] Booth A, Lowis C, Dean M, Hunter S, McKinley M (2013). Diet and physical activity in the self-management of type 2 diabetes: barriers and facilitators identified by patients and health professionals. Prim Health Care Res Dev.

[8] Pan X, Li G, Hu Y, Wang J, Yang W, An Z, Hu Z, Lin J, Xiao J, Cao H, Jiang X, Jiang Y, Wang JP, Zheng H, Zhang H, Bennet P, Howard B (1997). Effects of diet and exercise in preventing NIDDM in people with impaired glucose tolerance. The Da Qing IGT and Diab Study Diab Care.

[9] Yoon U, Kwok L, Magkidis A (2013). Efficacy of lifestyle interventions in reducing diabetes incidence in patients with impaired glucose tolerance: a systematic review of randomized controlled trials. Metabolism.

[10] Bischoff A, Ekoe T, Perone N, Slama S, Loutan L (2009). Chronic disease management in Sub-Saharan Africa: whose business is it?. Inter J Environ Res Public Health.

[11] Bradley H, Puoane T (2007). Prevention of hypertension and diabetes in an urban setting in South Africa: participatory action research with community health workers. Ethn Dis.

[12] Bray G, Look M, Ryan D (2013). Treatment of the obese patient in primary care: targeting and meeting goals and expectations. Postgrad Med.

[13] Health Quality Ontario (2009). Behavioural interventions for type 2 diabetes: an evidence-based analysis. Ont Health Technol Assess Ser.

[14] Angermayr L, Melchart D, Linde K (2010). Multifactorial lifestyle interventions in the primary and secondary prevention of cardiovascular disease and type 2 diabetes mellitus–a systematic review of randomized controlled trials. Ann Behav Med.

[15] Gilstrap L, Malhotra R, Peltier-Saxe D, Slicas D, Pineda E, Culhane-Hermann C, Cook N, Fernandez-Golarz C, Wood M (2013). Community-based primary prevention programs decrease the rate of metabolic syndrome among socioeconomically disadvantaged women. J Womens Health (Larchmt) 2013.

[16] Penn L, Dombrowski S, Sniehotta F, White M (2013). Participants' perspectives on making and maintaining behavioural changes in a lifestyle intervention for type 2 diabetes prevention: a qualitative study using the theory domain framework. BMJ Open.

[17] Carmoi T, Verret C, Debonne J, Klotz E (2008). Management of type 2 diabetes in subsaharan Africa: update and perspective. Med Trop (Mars).

[18] Whyte SR (2012). Chronicity and control: framing 'noncommunicable diseases' in Africa. Anthropol Med.

[19] Lasky D, Becerra E, Boto W, Otim M, Ntambi J (2002). Obesity and gender differences in the risk of type 2 diabetes mellitus in Uganda. Nutrition.

[20] Mayega R, Guwatudde D, Makumbi F, Nakwagala F, Peterson S, Tomson G, Ostenson C (2013). Diabetes and pre-diabetes among persons aged 35 to 60 years in eastern Uganda: prevalence and associated factors. Plos One.

[21] Mayega RW, Makumbi F, Rutebemberwa E, Peterson S, Ostenson CG, Tomson G, Guwatudde D (2012). Modifiable socio-behavioural factors associated with overweight and hypertension among persons aged 35 to 60 years in eastern Uganda. PLoS One.

[22] Awah P, Kengne A, Fezeu L, Mbanya J (2008). Perceived risk factors of cardiovascular diseases and diabetes in Cameroon. Health Educ Res.

[23] Draper C, Nemutandani S, Grimsrud A, Rudolph M, Kolbe-Alexander T, de Kock L, Lambert E (2010). Qualitative evaluation of a physical activity-based chronic disease prevention program in a low-income, rural South African setting. Rural Remote Health.

[24] Rutebemberwa E, Katureebe S, Gitta S, Mwaka A, Atuyambe L (2013). Perceptions of diabetes in rural areas of Eastern Uganda. Curationis.

[25] Turyatemba J (2011). Iganga-Mayuge DSS at the Helm of Research. Makerere University School of Graduate Studies.

[26] Wiersma W (1995). Research methods in education: an introduction. Allyn and Bacon.

[27] WHO, IDF (2006). Definition and diagnosis of diabetes mellitus and intermediate hyperglycemia: report of a WHO/IDF consultation.

[28] Mayega RW, Guwatudde D, Makumbi F, Nakwagala FN, Peterson S, Tomson G, Ostenson CG (2013). Diabetes and pre-diabetes among persons aged 35 to 60 years in eastern Uganda: prevalence and associated factors. PLoS One.

[29] WHO (1995). Physial Status: The Use and Interpretation of Anthropometry: Report of a WHO Expert Committee.

[30] Miller W, Rollnick S (2002). Motivational Interviewing: Preparing People to Change.

[31] Witt DR, Lindquist R, Treat-Jacobson D, Boucher JL, Konety SH, Savik K (2013). Motivational interviewing to reduce cardiovascular risk in African American and Latina women. West J Nurs Res.

[32] Carter P, Khunti K, Davies MJ (2012). Dietary recommendations for the prevention of type 2 diabetes: what Are they based on?. J Nutri Metab.

[33] Geneva W, WHO (2007). Increasing Physical Activity: A Guide for population level approaches.

[34] Graneheim U, Lundman B (2004). Qualitative content analysis in nursing research: concepts, procedures and measures to achieve trustworthiness. Nurse Educ Today.

[35] Nelson B, Chung P, DuPlessis H, Flores L, Ryan G, Kataoka S (2011). Strengthening families of children with developmental concerns: parent perceptions of developmental screening and services in Head Start. Ethn Dis.

[36] Baldwin AS, Rothman AJ, Vander Weg MW, Christensen AJ (2013). Examining causal components and a mediating process underlying self-generated health arguments for exercise and smoking cessation. Health Psychol: Off J Division of Health Psychol Am Psychol Assoc.

[37] Khattab MS, Abolfotouh MA, Alakija W, Al-Humaidi MA, Al-Wahat S (1999). **Risk factors of coronary heart disease**: **attitude and behaviour in family practice in Saudi Arabia**. *Eastern Mediterranean health journal = La revue de sante de la Mediterranee orientale = al-*. Majallah al-sihhiyah li-sharq al-mutawassit.

[38] Silagy C, Muir J, Coulter A, Thorogood M, Roe L (1993). Cardiovascular risk and attitudes to lifestyle: what do patients think?. BMJ (Clin Res ed).

[39] Jones E, Appel S, Eaves Y, Moneyham L, Oster R, Ovalle F (2012). Cardiometabolic risk, knowledge, risk perception, and self-efficacy among American Indian women with previous gestational diabetes. J Obstet Gynecol Neonatal Nurs.

[40] Pawlak R, Colby S (2009). Benefits, barriers, self-efficacy and knowledge regarding healthy foods; perception of African Americans living in eastern North Carolina. Nutrition Res Pract.

[41] Holdsworth M, Gartner A, Landais E, Maire B, Delpeuch F (2004). Perceptions of healthy and desirable body size in urban Senegalese women. Int J Obes Relat Metab Disord.

[42] Stern R, Puoane T, Tsolekile L (2010). An exploration into the determinants of noncommunicable diseases among rural-to-urban migrants in periurban South Africa. Preventing chronic Dis.

[43] Rguibi M, Belahsen R (2006). Fattening practices among Moroccan Saharawi women. Eastern Mediterranean health journal = La revue de sante de la Mediterranee orientale = al-Majallah al-sihhiyah li-sharq al-mutawassit.

[44] Duda R, Jumah N, Hill A, Seffah J, Biritwum R (2006). Interest in healthy living outweighs presumed cultural norms for obesity for Ghanaian women. Health Qual Life Outcomes.

[45] Lucas A, Murray E, Kinra S (2013). Heath beliefs of UK South Asians related to lifestyle diseases: a review of qualitative literature. J Obes.

[46] Johnson M, Everson-Hock E, Jones R, Woods HB, Payne N, Goyder E (2011). What are the barriers to primary prevention of type 2 diabetes in black and minority ethnic groups in the UK? A qualitative evidence synthesis. Diabetes Res Clin Pract.

[47] Orleans CT (2000). Promoting the maintenance of health behavior change: recommendations for the next generation of research and practice. Health Psychol: Off J of the Division of Health Psychol, Am Psychol Assoc.

[48] Muhihi A, Gimbi D, Njelekela M, Shemaghembe E, Mwambene K, Chiwanga F, Malik V, Wedick N, Spiegelman D, Hu F, Willet W (2013). Consumption and acceptability of whole grain staples for lowering markers of diabetes risk among overweight and obese Tanzanian adults. Global Health.

[49] Kiawi E, Edwards R, Shu J, Unwin N, Kamadjeu R, Mbanya JC (2006). Knowledge, attitudes, and behavior relating to diabetes and its main risk factors among urban residents in Cameroon: a qualitative survey. Ethnicity & Dis.

[50] Bishop J, Irby M, Isom S, Blackwell C, Vitolins M, Skelton J (2013). Diabetes prevention, weight loss, and social support: program participants' perceived influence on the health behaviors of their social support system. Fam Community Health.

[51] Miller S, Marolen K (2012). Physical activity-related experiences, counseling expectations, personal responsibility, and altruism among urban African American women with type 2 diabetes. Diab Educator.

[52] Bhanji S, Khuwaja AK, Siddiqui F, Azam I, Kazmi K (2011). Underestimation of weight and its associated factors among overweight and obese adults in Pakistan: a cross sectional study. BMC Public Health.

[53] Ettarh R, Van de Vijver S, Oti S, Kyobutungi C (2013). Overweight, obesity, and perception of body image among slum residents in nairobi, kenya, 2008–2009. Preventing chronic Dis.

[54] Kruger HS, Puoane T, Senekal M, van der Merwe MT (2005). Obesity in South Africa: challenges for government and health professionals. Public Health Nutr.

[55] Brenner B (2002). Implementing a community intervention program for health promotion. Soc Work Health Care.

[56] Rutebemberwa E, Kallander K, Tomson G, Peterson S, Pariyo G (2009). Determinants of delay in care-seeking for febrile children in eastern Uganda. Tropical Med Intern Health : TM & IH.

[57] El Zeiny NA (2000). Health and lifestyle survey: community's attitudes to health and barriers toward lifestyle change. J Egyptian Public Health Assoc.

[58] Cooper M, Harding S, Mullen K, O'Donnell C (2012). 'A chronic disease is a disease which keeps coming back…it is like the flu': chronic disease risk perception and explanatory models among French- and Swahili-speaking African migrants. Ethnicity & health.

[59] Jones EJ, Roche CC, Appel SJ (2009). A review of the health beliefs and lifestyle behaviors of women with previous gestational diabetes. J Obstet Gynecol Neonatal Nurs.

[60] Netto G, Bhopal R, Lederle N, Khatoon J, Jackson A (2010). How can health promotion interventions be adapted for minority ethnic communities? Five principles for guiding the development of behavioural interventions. Health Promot Int.

[61] Rothman AJ (2000). Toward a theory-based analysis of behavioral maintenance. Health Psychol: off J of the Division of Health Psychol Am Psychol Assoc.

[CR62] The pre-publication history for this paper can be accessed here:http://www.biomedcentral.com/1471-2458/14/864/prepub

